# Lentinan-Based Oral Nanoparticle Loaded Budesonide With Macrophage-Targeting Ability for Treatment of Ulcerative Colitis

**DOI:** 10.3389/fbioe.2021.702173

**Published:** 2021-08-27

**Authors:** Meisi Lin, Lingling Dong, Qiyan Chen, Haiting Xu, Xiaoqin Han, Ruifeng Luo, Xiulan Pu, Shanshan Qi, Wenbiao Nie, Meilin Ma, Yitao Wang, Fei Gao, Jinming Zhang

**Affiliations:** ^1^ State Key Laboratory of Southwestern Chinese Medicine Resources, Pharmacy School, Chengdu University of Traditional Chinese Medicine, Chengdu, China; ^2^ Affiliated Hospital, Chengdu University of Traditional Chinese Medicine, Chengdu, China; ^3^ Sichuan Provincial Acupuncture School, Chengdu, China; ^4^ State Key Laboratory of Quality Research in Chinese Medicine, Institute of Chinese Medical Sciences, University of Macau, Macao, China

**Keywords:** lentinan, budesonide, macrophage-target, oral nanoparticles, ulcerative colitis

## Abstract

Ulcerative colitis (UC) is a global, chronic, and refractory disease. Corticosteroids are first-line drugs for the treatment of UC but also cause adverse side effects. Budesonide (BUD), a corticosteroid with relatively low side effects, has been approved by the Food and Drug Administration for use as enteric capsules (Entocort EC) for the treatment of inflammatory bowel disease (IBD). However, this formulation lacks specific targeting ability to UC lesions. Herein, we describe the development of an advanced macrophage-targeted oral lentinan (LNT)–based nanoparticles (NPs) loaded BUD for treatment of UC. Briefly, LNT was used as a food source and natural carrier to load BUD by a simple solvent evaporation method to form LNT/BUD-NPs. LNT showed good loading capacity with high encapsulation and loading efficiencies to BUD of approximately 92.19 and 9.58%, respectively. Evaluation of the gastric stability of LNT/BUD-NPs indicated that LNT could effectively protect BUD from gastric acid and digestive enzymes. The release behavior and transmission electron microscopy image of LNT/BUD-NPs in the intestinal content of mice confirmed that intestinal flora can promote BUD release from LNT. Moreover, evaluation of cellular uptake showed that LNT/BUD-NPs could specifically target macrophages and enhance their uptake rate *via* the Dectin-1 receptor. In biodistribution studies, LNT/BUD-NPs were able to efficiently accumulate in the inflamed colon of mice. As expected, LNT/BUD-NPs could significantly alleviate inflammation by inhibiting the TLR4/MyD88/NF-κB signaling pathway. Therefore, LNT/BUD-NPs have the advantages of good gastric stability, release mediated by mouse intestinal content, macrophage-targeting, and anti-UC effects. These advantages indicate LNT-based NPs are a promising oral drug delivery system for UC therapy.

## Introduction

Ulcerative colitis (UC) is a common inflammatory bowel disease (IBD), which is defined by mucosal inflammation involving mainly the colon and the rectum (Luo et al., 2021). The United States and Europe are recognized as regions with a high incidence of UC (Armuzzi et al., 2020). However, recently, a clear increase in UC was also observed in some parts of Asia (Ng et al., 2019); for example, in Taiwan, the incidence rate increased from 2.1/100,000 in 2001 to 12.8/100,000 in 2015 (Yen et al., 2019). Clinically, UC is a chronic illness requiring long-term treatment (Rubin et al., 2019). The common therapeutic options are steroidal or hormonal drugs, such as aminosalicylic acid and glucocorticoids, which are used to control the disease and to maintain remission (Zhao et al., 2017).

Budesonide (BUD) is a typical glucocorticoid, which is used to treat IBD, arthritis, and asthma (Ali et al., 2016). Compared with other glucocorticoids, BUD has fewer side effects by oral administration, which is due to 90% of BUD being inactivated in the liver before reaching the systemic circulation, with only 10% BUD entering the systemic circulation (Miehlke et al., 2018). In fact, Entocort EC, a BUD enteric capsule formulation, was approved by the Food and Drug Administration in 2001 for the treatment of Crohn’s disease leading to a positive outcome (McKeage and Goa, 2002). However, enteric capsules are more suitable for Crohn’s disease which usually involves the entire intestine, rather than UC which is located at the distal part of the colon (Edsbacker and Andersson, 2004). In fact, Entocort EC was used to treat UC by retention enema instead of oral administration, leading to poor patient compliance. Therefore, based on the application of BUD in UC treatment, it is necessary to develop an effective and specific oral system for the delivery of BUD to UC lesions.

Recently, some studies have tried using oral nanodrug delivery systems to encapsulate BUD for UC treatment. However, most carriers are synthetic materials, which may have some toxicity limiting further application (Krishnamachari et al., 2007; Naeem et al., 2015; Zhou and Qian, 2018; Naeem et al., 2015). Lentinan (LNT), a natural, food-based polysaccharide (Zhang et al., 2011), is safe for oral administration. Four promising advantages of BUD delivery for UC treatment are as follows. First, LNT is mainly constituted by β-glucan which can specifically bind to the Dectin-1 receptor on the surface of macrophages (Lee and Kim, 2014). Therefore, LNT could help BUD to demonstrate targeting ability to inflammatory tissue with abundant macrophages. Second, based on the polysaccharide structure, LNT has the promising ability to remain stable within the physiological environment of the stomach, avoiding damage by gastric acid pH and digestive enzymes to BUD (Gani et al., 2018; Vetvicka et al., 2020). Third, degradation of LNT usually relies on the metabolites of intestinal microflora, such as β-glucanase (Hemsworth et al., 2016). Hence, the massively growing flora around UC lesions could increase the degradation rate of LNT, releasing loaded BUD from LNT target UC lesions. Last, LNT also has an independent pharmacological effect on UC, which may exhibit synergy with BUD (Mitamura et al., 2000). Unfortunately, few studies have used LNT to encapsulate drugs for UC treatment. Therefore, LNT could be a promising carrier in loading BUD for targeting macrophages for treatment of UC by oral administration.

Herein, a simple solvent evaporation method was used to form LNT/BUD-NPs. The loading capacity of LNT and the physicochemical characterization of NPs were evaluated. The gastric protection ability, intestinal content-induced releasing behavior, and macrophage-targeting efficacy of LNT/BUD-NPs are highlighted in this study. The targeting ability and anti-UC effects involving the promising TLR4/NF-κB pathway were evaluated *in vivo*.

## Materials and Methods

### Materials

The animal experiments were approved by the ethics committee of the Chengdu University of Traditional Chinese Medicine (CDUTCM, permit CDU2019S121), and all animal experiments were conducted in strict accordance with the Guidelines for the Care and Use of Laboratory Animals of the Ministry of Science and Technology of China. Male BALB/c mice (22–25 g) were obtained from SPF (Beijing) Biotechnology Co., Ltd. (Beijing, China).

LNT were purchased from DESITE Biological Technology Co., Ltd. (>98%, DST200405085, Sichuan, China), BUD were purchased from DESITE Biological Technology Co., Ltd. (>98%, DSTSB027201, Sichuan, China), and Dimethyl sulfoxide (DMSO) was purchased from Macklin Biochemical Co., Ltd. (Shanghai, China). Coumarin-6 (C_6_) was obtained from Aladdin Reagent Company (Shanghai, China). Near-infrared lipophilic carbocyanine dye 1,10-dioctadecyltetramethyl indotricarbocyanine iodide (DiR, MW = 1,013.39) was purchased from Mellon Biological Technology Co., Ltd. (Dalian, China) and Dextran sodium sulfate (DSS, 36–50 kDa) was obtained from MP Biomedicals Inc. (California, United States). Hoechst 33342 was provided by Suzhou Yuheng Biotechnology Co., Ltd. (Suzhou, China). Inducible nitric oxide synthase (iNOS) kit was obtained from Elabscience Biotechnology Co., Ltd. (Wuhan, China). Interleukin (IL)-1β, tumor necrosis factor-alpha (TNF-α), IL-6 kits and anti-myeloid differentiation factor 88 (MyD88), anti-NF-κB p65, and anti-TLR4 antibodies were obtained from MultiScience (Lianke) Biotech Co., Ltd. (Hangzhou, China). Leukemic mouse macrophage cells (RAW264.7) were purchased from the American Type Culture Collection (Manassas, United States).

### Preparation of LNT/BUD-NPs

A simple solvent evaporation method with some modifications was used to prepare LNT/BUD-NPs. In brief, 50 mg LNT was dissolved in 20 ml deionized water. Meanwhile, 5 mg BUD was dissolved in 2 ml acetone to prepare BUD solution. In order to improve solubility, the above solution and organic phase were sonicated by a probe sonicator (Sonicator XL, Misonix, Melville, NY, United States) for 5 min. Then, the BUD solution was dropped into the LNT solution at a slow, uniform rate with stirring. To prepare the LNT/BUD-NPs, this mix solution was stirred at 300 rpm at 25°C for 12 h to eliminate all acetone. Finally, LNT/BUD-NPs were stored at 4°C for further experiments.

### Physicochemical Characterization of NPs

Dynamic light scattering (DLS) with a Particle Analyzer Litesizer 500 (Anton Paar, Austria) was used to measure the average hydrodynamic particle size, polydispersity index (PDI), and zeta potential of LNT/BUD-NPs. Average values were measured separately from three separate trials based on different batches of NPs.Transmission Electron Microscopy (TEM, JEM 1200X, JEOL, Japan) was used to analyze the morphology of LNT/BUD-NPs.

Transmission electron microscopy (TEM, JEM 1200X, JEOL, Japan) was used to analyze the morphology of the LNT/BUD-NPs (Gao et al., 2017). The diluted sample was dropped onto the surface of the carbon-coated copper grid; after 5 min, a filter paper was then used to remove the excess solution. Based on a published article, the measured sample was negatively stained with uranyl acetate and imaged using TEM.

X-ray diffraction (XRD) spectra of Free LNT, Free BUD, Free LNT and BUD physical mixture (MIX), Blank-NPs, and LNT/BUD-NPs were detected by X-ray diffractometry (D8 Advance, BRUKER, Germany), operating at 40 kV and 40 mA and scanning at a rate of 6° per min from 5° to 90°(Luo et al., 2021).

A Fourier transform infrared spectrometer (IR Tracer-100, Shimadzu, Japan) was used to record the infrared (IR) spectra of Free LNT, Free BUD, MIX, Blank-NPs, and LNT/BUD-NPs. Wave number range was set from 4,000 to 400 cm^−1^, and the spectra were recorded with an average of 16 scans at 4 cm^−1^ resolution. In brief, samples were mixed with KBr, and the mixture was then pressed into a pancake for further evaluation (Gou et al., 2018).

Encapsulation efficiency (EE, %) and loading efficiency (LE, %) of BUD were evaluated by high-performance liquid chromatography (HPLC) (LC-45202–46, SHIMADZU, Japan), equipped with a C_18_ column (250 × 4.6 mm). The ultraviolet (UV) detector wavelength was set at 244 nm, and the mobile phase was a combination of methanol/deionized water (72/28, v/v) at a flow rate of 1 ml/min (Zhou et al., 2020). Before HPLC analysis, methanol was used to destroy the structure of the NPs and dissolve the BUD. The drug EE and LE were calculated by following equations:

1) EE (%) = Amount of RH loaded/Amount of RH added × 100%
2) LE (%) = Amount of BUD loaded/Total amount of NPs harvested × 100%

### Gastric Stability of LNT/BUD-NPs

The previously published method was used to evaluate the drug’s stability in simulated gastric fluid (SGF) encapsulated in LNT/BUD-NPs (Luo et al., 2020). Briefly, 1 ml BUD loaded LNT/BUD-NPs were immersed in 9 ml SGF (pH 2, containing 1 mg/ml pepsin) and then incubated in a shaking bath at constant temperature (37°C; 100 rpm) for 4 h. The average hydrodynamic particle size was measured using DLS with a Particle Analyzer Lite sizer 500. The morphology of LNT/B-NPs after processed with SGF was measured using TEM.

### Intestinal Stability and Drug Release Profiles of LNT/BUD-NPs

The stability of LNT/BUD-NPs was evaluated in 2% mouse intestinal content. Briefly, LNT/BUD-NPs was incubated for 12 h in PBS solution and 2% mouse intestinal content, respectively, and then evaluated for size distribution and TEM morphology for further analysis (Zhou et al., 2020).

BUD release profiles from Free BUD, LNT/BUD-NPs were examined by dialysis method. 3 ml Free BUD and LNT/BUD-NPs were added into dialysis bags separately (molecular weight cutoff value 1,000 Da). The bags containing Free BUD and LNT/BUD-NPs were immersed in 30 ml PBS medium (pH 7.4) for further evaluation of release profiles. Meanwhile, another dialysis bag containing 3 ml LNT/BUD-NPs was soaked in 30 ml PBS (pH 7.4) containing 2% mouse intestinal content. The three groups of samples were shaken at 37°C at a rate of 100 rpm. 1 ml outer solution was collected at defined time points (0, 0.5, 1, 2, 3, 4, 6, 8, 10, 12, 16, 20, and 24 h), and 1 ml of fresh release medium was added. As mentioned above, cumulative release (%) was measured by HPLC. The morphology of LNT/BUD-NPs after processing with 2% mouse intestinal content was also evaluated by TEM.

### Cell Culture

RAW264.7 macrophages were cultured in MEM/DMEM medium supplemented with 1% streptomycin, penicillin (Thermo Fisher Scientific, United States), and 10% fetal bovine serum (Thermo Fisher Scientific, Billerica, United States).

### Cellular Uptake Investigation

Cellular uptake is a key indicator of *in vitro* nanomedicine behavior. We used flow cytometry (FCM; NovoCyte; ACEA, San Diego, CA, United States) and confocal laser scanning microscopy (CLSM; TCS SP8 SR; Leica, Weztlar, Germany) to measure efficiency of cellular uptake (Gou et al., 2019). Since BUD does not fluoresce significantly, C_6_ was used instead of BUD as a fluorescent probe. Raw264.7 macrophages were used for the assessment of cellular uptake.

CLSM was used for the qualitative analysis of cellular uptake. The confocal dishes were used to culture cells at a concentration of 10^5^ cells/ml for 12 h. RAW264.7 macrophage cells were incubated with Free C_6_ and LNT/C_6_-NPs at a concentration of 100 ng/ml for 4 h. Moreover, in order to confirm the targeting ability of LNT to macrophages, cells were pretreated with laminarin (1 mg/ml) to prebind the Dectin-1 receptors (Dulal et al., 2018), and then incubated with LNT/C6-NPs under the same dosage. After that, the cells were rinsed with cold PBS, fixed in 4% paraformaldehyde solution, and stained with Hoechst 33342; CLSM was used to observe cells.

FCM was used for the quantitative analysis of cellular uptake. Raw264.7 macrophage cells were seeded in 6-well plates and cultured for 12 h until adherence; cells were then cultured at 37°C for further study. Regarding differences in cellular uptake among different preparations, the cells were incubated with Free C_6_ and LNT/C_6_-NPs preparations (100 ng/ml) for 4 h. Simultaneously, cells were pretreated with laminarin and incubated with the LNT/C_6_-NPs group, which was compared with the non-pretreated LNT/C6-NPs group. For the dosage-dependent investigation, cells were incubated with LNT/C_6_-NPs in different concentrations (6.25, 12.5, 25, 50, and 100 ng/ml). For the time-dependent investigation, cells were incubated with LNT/C_6_-NPs (100 ng/ml) at different time points (0.25, 0.5, 1, 2, and 4 h). In all experiments, untreated RAW264.7 macrophages were used as controls. After incubation, cells were rinsed with cold PBS, collected by centrifugation, and re-suspended in PBS. Finally, the cell suspension was analyzed using FCM.

### *In Vivo* Biodistribution Evaluation

To evaluate the distribution of different preparations in the intestine after oral administration, the near-infrared dye, DiR, was used as a fluorescent probe. The UC mouse model was induced by drinking 3% DSS solution for 7 days (Wang et al., 2017); mice were then separated into two groups (Free DiR and LNT/DiR-NPs). The two groups were given the corresponding preparations at a DiR concentration of 0.5 mg per kg. After oral administration for 3, 6, 12, and 24 h, the mice were photographed using a Caliper Life Sciences LIVIS Lumina Series (PerkinElmer, MA, United States). At the end point, mice were sacrificed to obtain the intestine and colon; after that, they were also photographed.

### *In Vivo* Therapeutic Evaluation

The DSS-induced UC mouse model was used to evaluate the *in vivo* anti-UC efficiency of BUD/LNT-NPs. Mice were randomly divided into six groups (*n* = 6 for each group) as follows: (1) Control group, (2) Model group, (3) Free BUD group, (4) LNT group, (5) MIX group, and (6) BUD/LNT-NPs group. As previously described, mice were marked on day 0 and their original weight was recorded. Except for the Control group which received only saline, the other groups drank 3% DSS solution to establish the UC mice model from day 0 to day 7. From day 4 to day 11, UC mice were treated by the different formulas (Beloqui et al., 2013), and the Model group received the equivalent amount of saline. During the entire experiment, daily changes in body weight, consistency of feces, and stool bleeding were recorded. Disease activity index (DAI) was a typical indicator in anti-UC evaluation, which was calculated by the sum of stool bleeding (0–4), consistency of feces (0–4), and weight loss (0–4) (Ali et al., 2016). On the last day, mice were anesthetized and euthanized, and the colon and major organs (heart, liver, spleen, lung, and kidney) were collected. The organs and colon were fixed in 4% formalin, embedded in paraffin, sectioned into slices (5 μm), and stained with hematoxylin and eosin (H&E). Inflammatory cytokines in colon tissue, including IL-1β, IL-6, iNOS, and TNF-α, were assessed by ELISA kits. Western blot was used to detect the expression of colon proteins, including TLR4, TRAF6, MyD88, IKKα, and NF-κB p65. Primary antibodies against TLR4, TRAF6, MyD88, IKKα, and NF-κBp65 were then incubated overnight at 4°C. Anti-β-actin antibodies were used as internal controls to ensure equal loading of samples. The obtained chemiluminescence signals were analyzed using ImageJ software.

### Statistical Analysis

All data were calculated by mean ± SD. All *in vitro* and *in vivo* experiments were analyzed by at least three independent tests under the same experimental conditions. Student’s *t*-test was used to analyze differences among the different groups. A *p-value* of <0.05 was considered significant.

## Results and Discussions

### Characterization of NPs

The characterization of LNT/BUD-NPs focused on size distribution, morphology, zeta potential, EE, LE, XRD, and FT-IR, the results were as follows.

**FIGURE 1:**
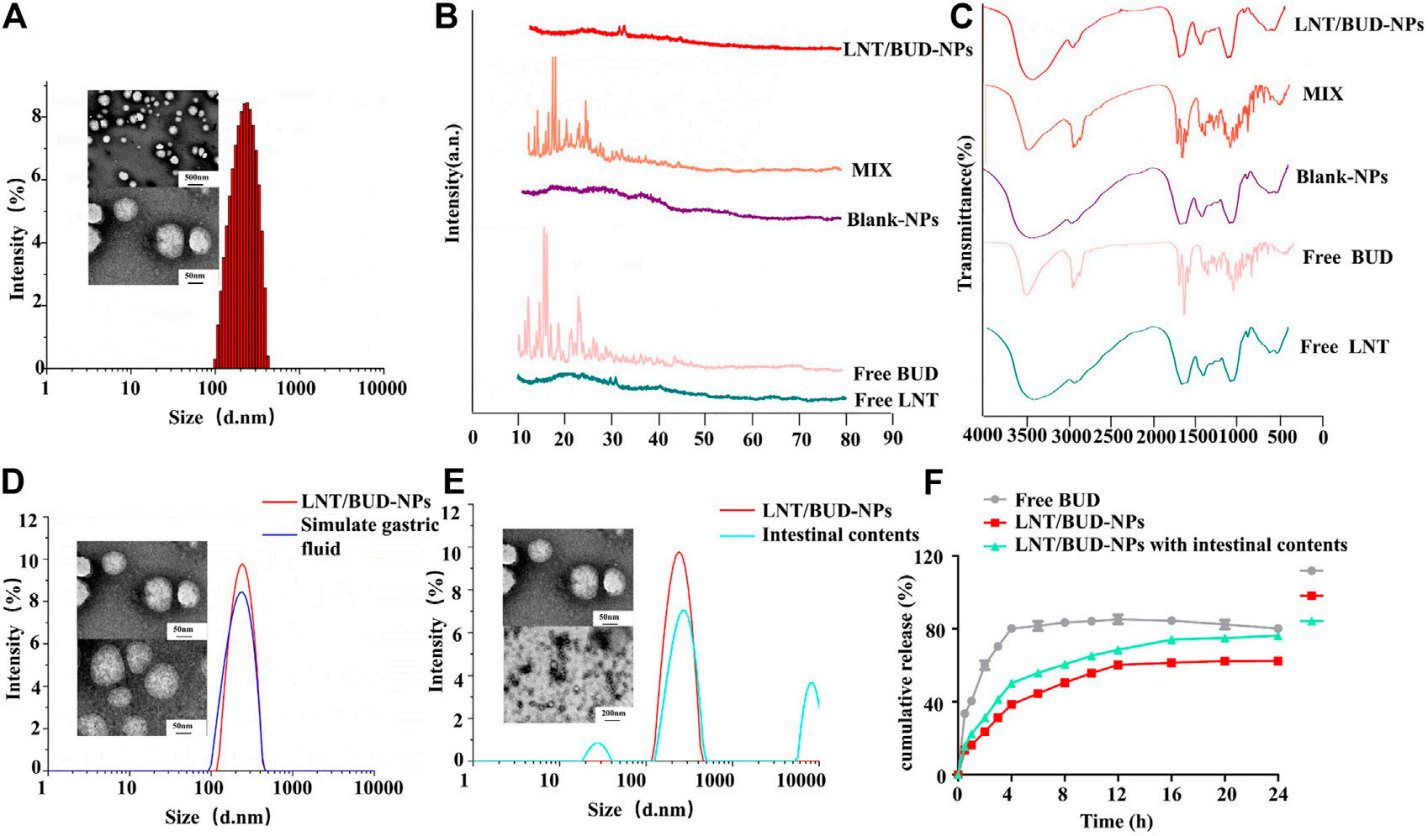
Characterization stability evaluation of NPs. **(A)** Size distribution and TEM images of LNT/BUD-NPs. **(B)** XRD spectrum of different formulas. **(C)** IR spectrum of different formulas. **(D)** Size distribution and TEM images of LNT/BUD-NPs in PBS or simulated gastric fluid. **(E)** Size distribution and TEM images of LNT/BUD-NPs in PBS or mice intestinal contents. **(F)** Release profile of Free BUD, LNT/BUD-NPs in PBS, and LNT/BUD-NPs in mouse intestinal contents.

Size distribution and TEM morphology images are shown in Figure 1A, the particle size of LNT/BUD-NPs was 261.15 ± 5.45 nm, with a relatively low PDI value (0.20 ± 0.01) and spherical structure, indicating a homogeneous size distribution. The zeta potential of the LNT/BUD-NPs was −3.10 ± 0.80 mV, the EE and LE of LNT/BUD-NPs was 92.19 ± 4.39% and 9.58 ± 0.66%, respectively. These data demonstrate that the LNT had a high drug loading capacity with a relatively narrow particle size distribution which may be suitable for delivery of BUD.

In order to confirm the crystallinity of BUD in LNT, XRD patterns and FT-IR spectra were studied. As presented in Figure 1B, representative XRD patterns of Free BUD show numerous sharp peaks between 10° and 25°, indicating the crystalline nature of BUD. However, these representative peaks were not present in LNT/BUD-NPs, indicating that a crystalline complex was not formed between BUD and its LNT matrix. Thus, these data demonstrate that crystal BUD was converted into amorphous molecules inside the LNT matrix (Gou et al., 2018).

As shown in Figure 1C, in the group of BUD, strong signals were detected at 1721, 1,667 cm^−1^ in the FT-IR spectrum, which could be assigned to the C=O group (C_17_) and the conjugated dihydrobenzoquinone carbonyl group (C_3_) (Amini et al., 2014; Buhecha et al., 2019). However, the two peaks were not present in LNT/BUD-NPs, suggesting that the BUD is not adsorbed on to the external surface of the LNT/BUD-NPs.

### Gastric Stability of NPs

The acid pH environment and digestive enzymes in the stomach may produce the negative influence on NPs during oral administration. To investigate whether LNT/BUD-NPs could safely survive the harsh gastric environment, SGF was used to evaluate LNT/BUD-NPs stability after oral gavage. After incubation for 4 h in SGF solution, the LNT/BUD-NPs solution was still homogeneous, with a particle size of 274 ± 16.38 nm and PDI of 0.25 ± 0.04, the TEM image also displayed an even and smooth morphology, which was similar to the untreated sample (Figure 1D). The results of particle size and TEM images both showed the LNT/BUD-NPs were stable in SGF, further showing the protective capability of LNT in the gastrointestinal environment, suggesting it may be a promising drug carrier and oral delivery system.

### *In Vitro* Drug Release Profile of LNT/BUD-NPs

To determine the intestinal stability of LNT/BUD-NPs with and without mouse intestinal content in buffer (pH 7.4), we evaluated both by size distribution and TEM. As shown in Figure 1E, after incubation with mouse intestinal content, the average size of LNT/BUD-NPs increased from 261.15 to 308.18 with three peaks, indicating the instability of LNT/BUD-NPs in intestinal contents. Similar outcomes were also observed in TEM, with the shape of LNT/BUD-NPs becoming nonuniform.

Release profiles of Free BUD and LNT/BUD-NPs were detected with and without mouse intestinal content, results are shown in Figure 1F, LNT/BUD-NPs were released more slowly than Free BUD. Briefly, approximately 80% of Free BUD was released after 4 h of incubation. Mouse intestinal content in the dissolution medium resulted in improved drug release from LNT/BUD-NPs at different time intervals in comparison to that carried out without using mouse intestinal content. In the absence of mice intestinal content, BUD from LNT/BUD-NPs released about 61% after 16 h, while the mouse intestinal content showed a catalytic effect on drug release, the BUD from LNT/BUD-NPs released up to approximately 74% after 16 h. Results of the *in vitro* release study revealed that intestinal content could break the structure of LNT/BUD-NPs promoting release, which may due to the metabolites of intestinal flora, such as β-glucanase which could degrade β-glucan of LNT leading directly to drug release (Zhang et al., 2011).

### Cellular Uptake Capacity of NPs

In order to study the uptake characteristics of LNT/BUD-NPs, Raw264.7 macrophages were used as a model. It is worth pointing out that no obvious fluorescence can be detected in BUD, the C_6_ probe was therefore used to replace BUD for uptake tracking.

**FIGURE 2:**
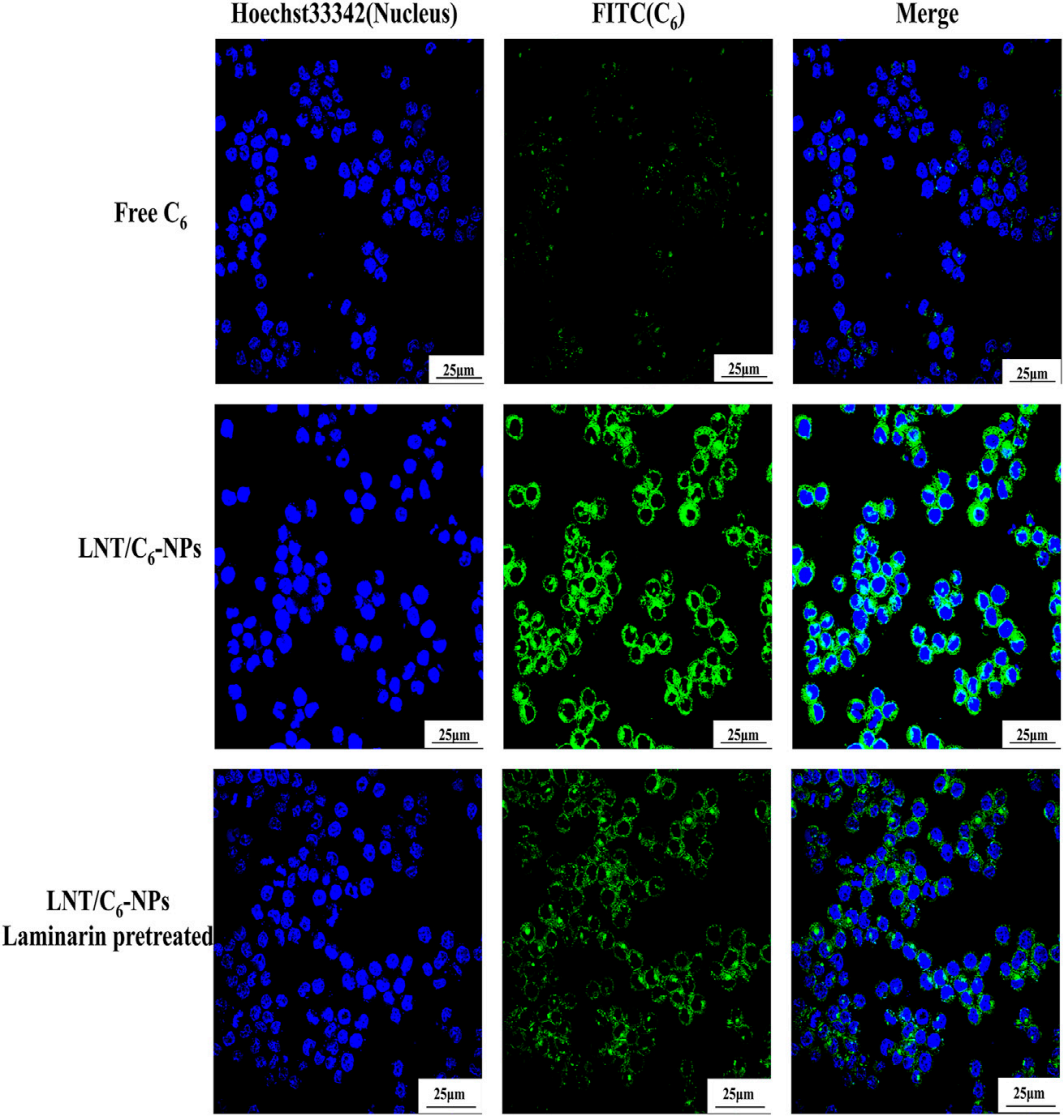
Qualitative analysis of macrophage cellular uptake capacity to Free C_6_ and LNT/C_6_-NPs.

CLSM is a common method for qualitative analysis of cellular uptake of NPs. As expected, poor green fluorescence was detected in Raw264.7 macrophages in the Free C_6_ group, while a stronger C_6_ signal was observed in the LNT/C_6_-NPs group (Figure 2), showing that LNT/C_6_-NPs were more easily taken up by macrophages than Free C_6_. However, after the Dectin-1 receptors on Raw264.7 macrophages were pretreated with laminarin, the uptake of LNT/C6-NPs by Raw264.7 macrophages decreased significantly, indicating that LNT could target Dectin-1 receptors of Raw264.7 macrophages so as to increase their efficiency of uptake.

**FIGURE 3:**
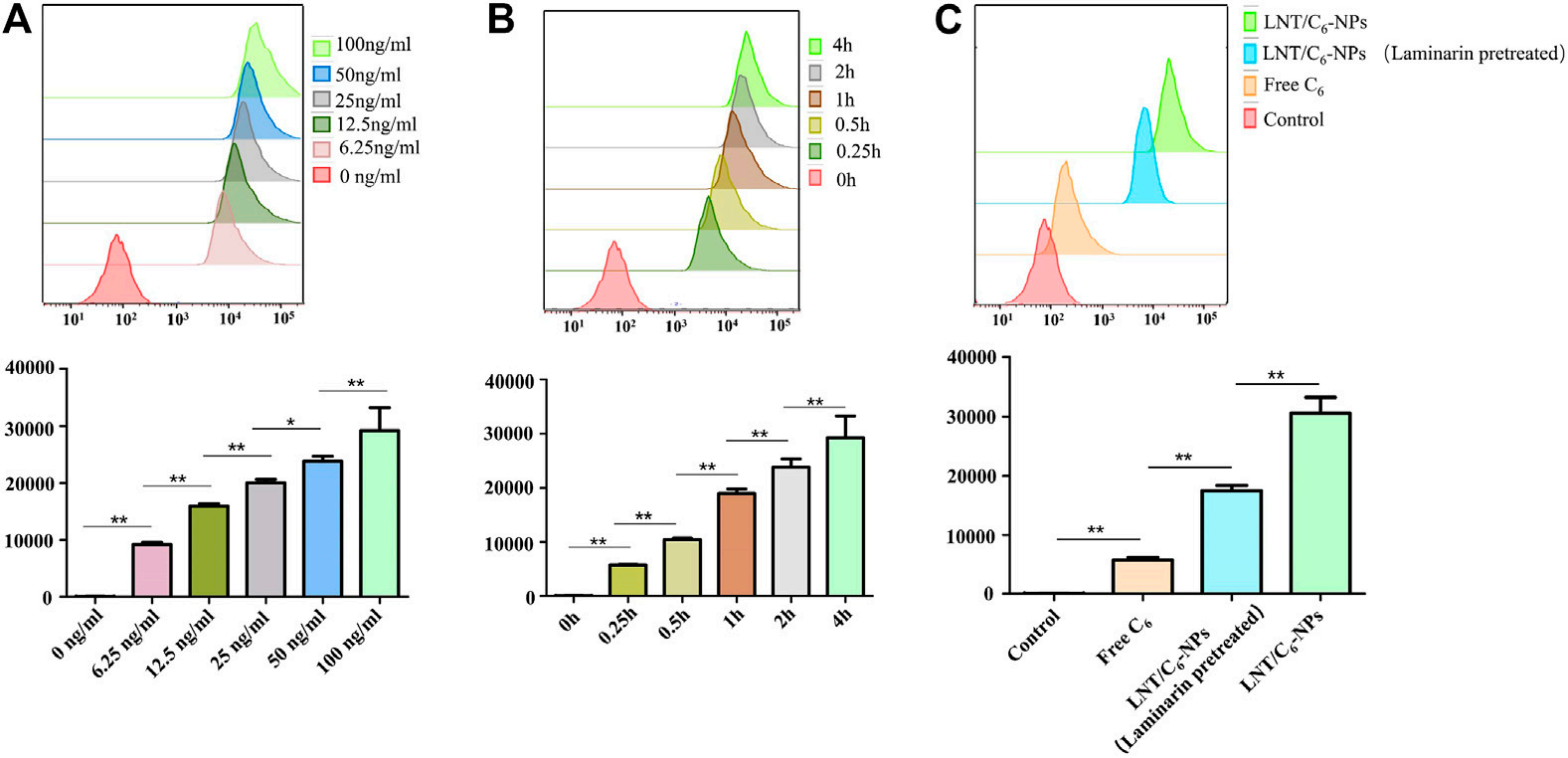
Quantitative analysis of macrophage cellular uptake capacity to different C_6_ formulas. **(A)** Quantitative measurement of LNT/C_6_-NPs uptake by Raw264.7 macrophages after incubation for 4 h at different concentrations (6.25, 12.5, 25, 50, and 100 ng/ml). **(B)** Quantitative measurement of LNT/C_6_-NPs uptake by Raw264.7 macrophages at different incubation times (0, 0.25, 0.5, 1, 2, and 4 h) with a C_6_ concentration of 100 ng/ml. **(C)** Quantitative analysis of macrophage cellular uptake capacity to Free C_6_ and LNT/C_6_-NPs. ^*^ *p* < 0.05; **p < 0.01, *n* = 3.

In addition, the project used FCM for quantitative analysis of NPs to confirm its uptake. As shown in Figures 3A,B, the signal of C_6_ in the LNT/C_6_-NPs group increased significantly with the concentration of C_6_ from 6.25 to 100 ng/ml (*p* < 0.01), indicating the concentration-dependent manner of LNT/C_6_-NPs in Raw264.7 macrophages. Similarly, the clear increased level of C_6_ in LNT/C_6_-NPs was also observed with time, increasing from 0 to 4 h (*p* < 0.01), indicating a time-dependent process in Raw264.7 macrophages. As shown in Figure 3C, after incubating Raw264.7 macrophages with Free C_6_ or LNT/C_6_-NPs at a concentration of 100 ng/ml for 4 h, the intensity of C_6_ in LNT/C_6_-NPs was clearly stronger than that of Free C_6_ (*p* < 0.01). However, after the Dectin-1 receptors on Raw264.7 macrophages were pretreated with laminarin, their fluorescence intensity was significantly decreased compared with the unpretreated group (*p* < 0.01). These results show that the uptake of C_6_ by Raw264.7 macrophages increased significantly after being encapsulated with LNT, and LNT had a strong targeting effect on Raw264.7 macrophages.

In general, both qualitative and quantitative analysis showed that LNT could increase the targeted uptake ability of macrophages to the NPs by the Dectin-1 receptors.

### *In Vivo* Biodistribution Evaluation

**FIGURE 4:**
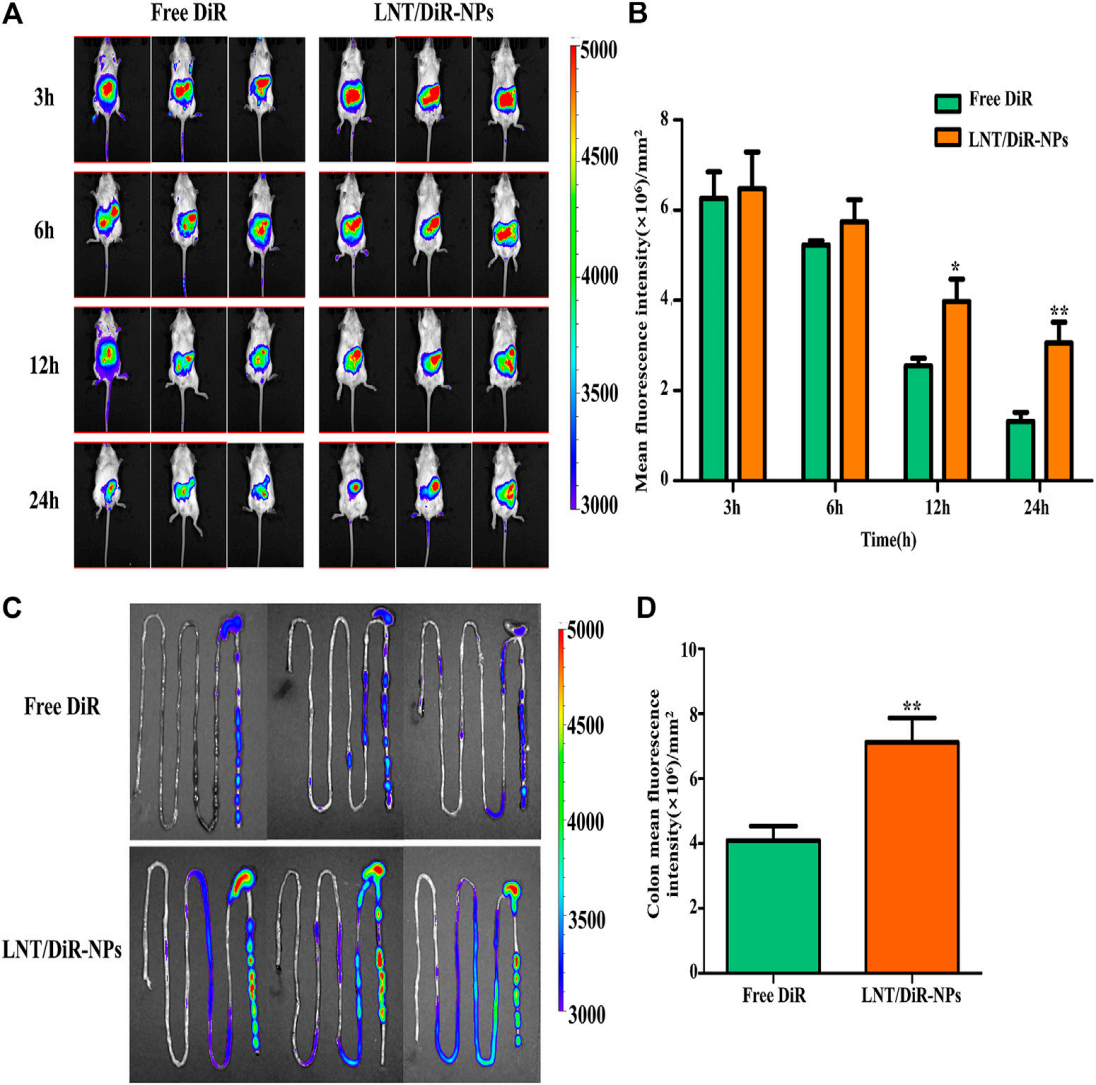
*In vivo* biodistribution evaluation of Free DiR and LNT/DiR-NPs to the DSS-induced UC mouse model. **(A)** Fluorescence images of mouse treated with Free DiR and LNT/DiR-NPs at different time points (3, 6, 12, and 24 h). **(B)** Histogram analysis of mouse fluorescence values between Free DiR and LNT/DiR-NPs treated mouse at different time points. **(C)** Fluorescence images of mouse intestine between Free DiR and LNT/DiR-NPs at 24 h. **(D)** Histogram analysis of intestinal fluorescence values between Free DiR and LNT/DiR-NPs at 24 h. ^*^ *p* < 0.05, **p < 0.01 vs. Free DiR, *n* = 6.

Accumulation of drugs around UC lesions is an important strategy in oral drug delivery. Therefore, in this study, DiR fluorescence probe was used to form the fluorescence labeling, then, IVIS was used to evaluate the targeting ability of LNT/DiR-NPs on colon. The DSS-induced UC mice were imaged at each time point after oral delivery in the different groups of DiR. As shown in Figures 4A,B, no obvious difference was observed among the different groups after administration for 3 h or 6 h. However, at 12 and 24 h, significant difference was detected between LNT/DiR-NPs and the Free DiR group (*p* < 0.05), which showed the better targeting ability of LNT/DiR-NPs. In addition, the mean fluorescence intensity of the colon was much higher than the LNT/DiR-NPs group and the Free DiR group (Figures 4C,D, *p* < 0.01). In summary, LNT could effectively enhance the targeting ability of loaded drugs to colon lesions due to the specific targeting of ligands of LNT to macrophages and the release of metabolites from flora in the lesion promoting BUD release from LNT.

### *In Vivo* Therapeutic Efficiency of LNT/BUD-NPs Against UC

The pathological characteristics of the DSS-induced UC mouse model are similar to human patients with UC and include loss of body weight, colon shortening, and hemafecia. For this reason, this model was employed to assess the therapeutic results of LNT/BUD-NPs against UC (Perse and Cerar, 2012).

**FIGURE 5:**
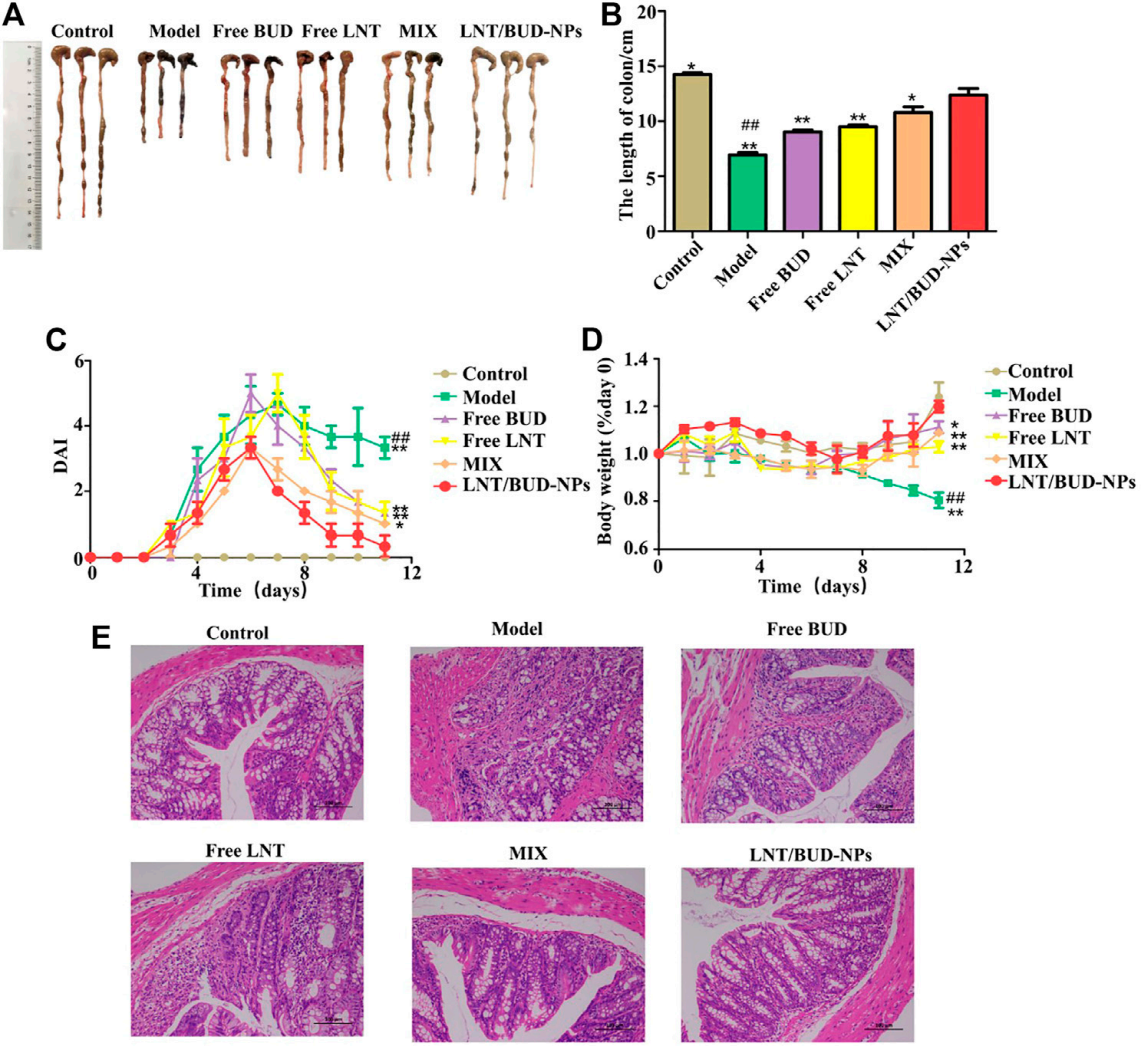
*In vivo* therapeutic efficiency of different formulas to DSS-induced UC in a mouse model. **(A)** Colon images of different formulas after treatment with different formulas, including Free LNT, Free BUD, MIX, and LNT/BUD-NPs. **(B)** Histogram analysis of colon length of mice from the different formulas. **(C)** Graph of DAI scores over time from the different formulas. **(D)** Graph of body weight over time from the different formulas. **(E)** H&E staining of colonic sections from the different formulas. ^*^ *p* < 0.05, ^**^ *p* < 0.01 vs. LNT/BUD-NPs; ^#^ *p* < 0.05, ##*p* < 0.01 vs. Model; *n* = 9.

As shown in Figure 5A, the colon length of the Model group was significantly shorter than the Control group (*p* < 0.01), being approximately 6.5 and 14 cm, respectively. Nevertheless, LNT/BUD-NPs could clearly inhibit colon shortening of DSS-induced UC, followed by the other three groups, MIX, Free LNT, and Free BUD (*p* < 0.05). Similar differences were observed for DAI scores. As shown in Figure 5C, DAI of the LNT/BUD-NPs group exhibited a decreasing trend and were lower than that of the DSS Model group. Meanwhile, on the last day, DAI scores of the LNT/BUD-NPs were clearly lower than the other three groups (*p* < 0.05), suggesting a better anti-inflammatory effect of LNT/BUD-NPs than Free BUD, Free LNT, and MIX. For body weight changes (Figure 5D), mice body weight slightly increased over time in the Normal group, while the body weight of the Model group induced by DSS significantly decreased by approximately 20% after 10 days (*p* < 0.01). LNT/BUD-NPs could significantly help the recovery of body weight loss compared with Free BUD, Free LNT, and MIX (*p* < 0.05), indicating the efficacy of LNT/BUD-NPs in helping mice with UC to recover. H&E staining was employed to further determine the therapeutic outcomes of LNT/BUD-NPs against UC. As shown in Figure 5E, there is no obvious inflammatory region in the colonic section of the Normal group. However, the Control group presented clear inflammation, including the accumulation of inflammatory cells and the disruption of colonic epithelia layer. MIX, Free LNT, and Free BUD treated groups displayed slight alleviation in the severity of inflammation compared with Control group. Simultaneously, the LNT/BUD-NPs treated group showed almost no accumulation of inflammatory cells in the colonic mucosa and maintained a similar morphology as the Control group, revealing the therapeutic efficiency of this approach. In addition, the H&E staining of different organs (heart, liver, spleen, lung, and kidney) sections from the different formulas were shown in Supplementary Figure 1, which indicated no obvious toxicity, were observed among each formula.

### *In Vivo* Inflammatory Cytokines Regulation

**FIGURE 6:**
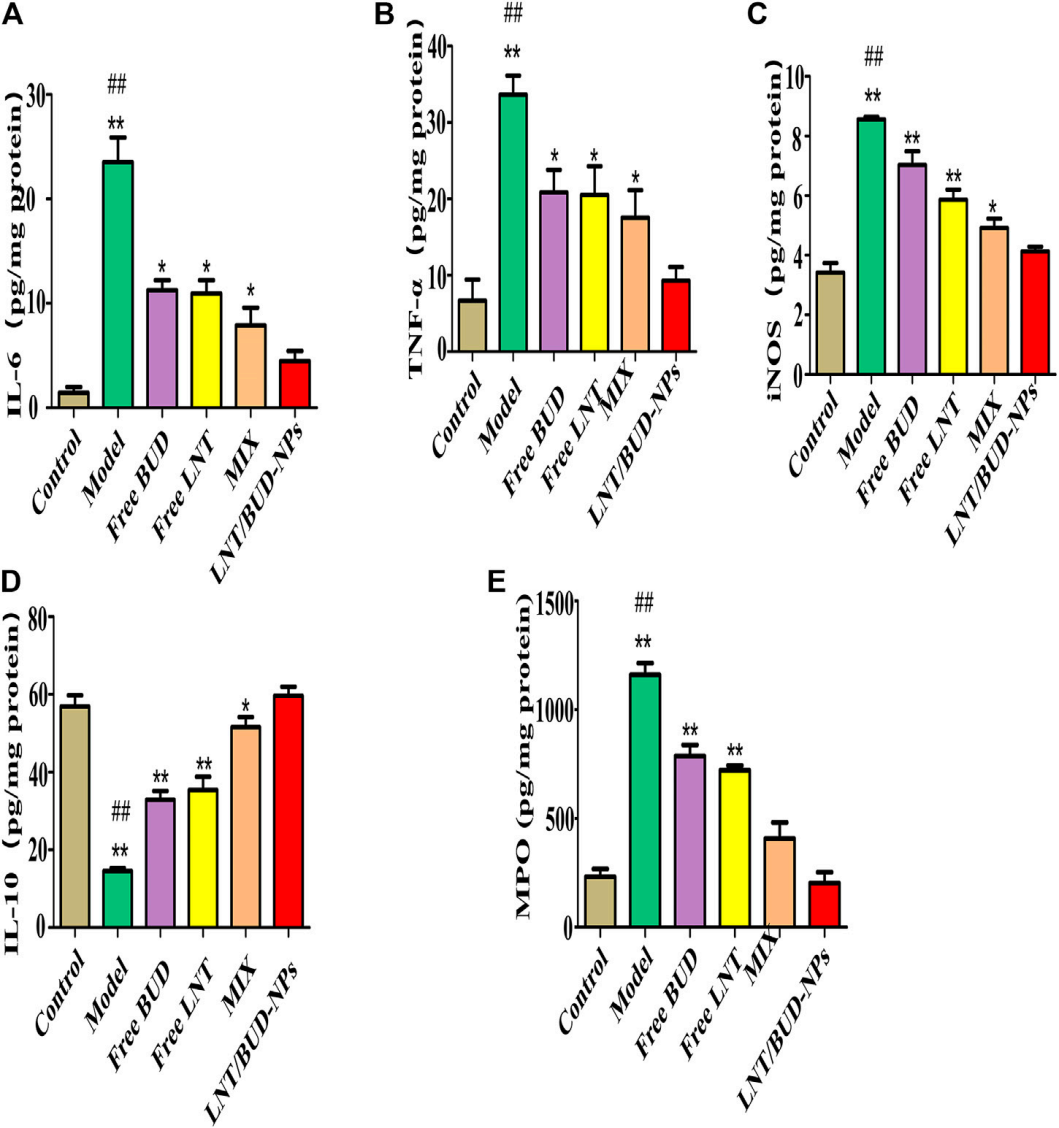
*In vivo* inflammatory cytokine evaluation of DSS-induced UC mice colon from the different formulas. **(A–E)** Histogram analysis of IL-6, TNF-α, iNOS, IL-10, and MPO level in colon among different formulas. ^*^ *p* < 0.05, **p < 0.01 vs. LNT/BUD-NPs; ^#^ *p* < 0.05, ^##^ *p* < 0.01 vs. Model; *n* = 9.

Inflammatory cytokines are an important index in anti-UC treatment. Therefore, iNOS, IL-6, TNF-α, and IL-10 were analyzed in this study (Liu et al., 2018). As shown in Figures 6A–D, compared with the Control group, the expression of iNOS, IL-6, and TNF-α were increased significantly while the expression of IL-10 decreased significantly in the Model group (*p* < 0.01), indicating the efficacy of DSS in inducing UC inflammation. After treatment with different formulas, all of the cytokines showed positive changes. Especially for the LNT/BUD-NPs group, where expression of iNOS, IL-6, and TNF-α was higher than that of the other three groups, including Free LNT, Free BUD, and MIX. This indicates the best anti-UC efficacy is mediated by LNT/BUD-NPs (*p* < 0.01). A similar outcome was observed for IL-10 expression. Moreover, MPO, is a significant indicator of inflammation in UC, which could be produced by macrophages and granulocytes (Aratani, 2018). As shown in Figure 6E, after treatment with LNT/BUD-NPs, the concentration of MPO was significantly decreased in contrast with the other four groups (*p* < 0.05), and the values were almost the same as the Control group. In summary, the results of the inflammatory cytokine analysis indicate that LNT/BUD-NPs show the most effective anti-inflammatory treatment for UC.

### *In Vivo* Anti-Inflammation by Inhibiting TLR4/NF-κB Signaling Pathway

TLR4 is one of the innate immune receptors and plays a vital role in the activation of the downstream inflammation signaling pathway of NF-κB through recruitment of the adaptor protein MyD88 and further, mediates secretion of inflammatory cytokines, such as TNF-α, IL-1β, IL-6, and iNOS (Liu et al., 2014). In order to verify the ability of LNT/BUD-NPs to alleviate inflammation in UC through the TLR4/NF-κB pathway, Western blot analysis were applied, the results were as follows.

**FIGURE 7:**
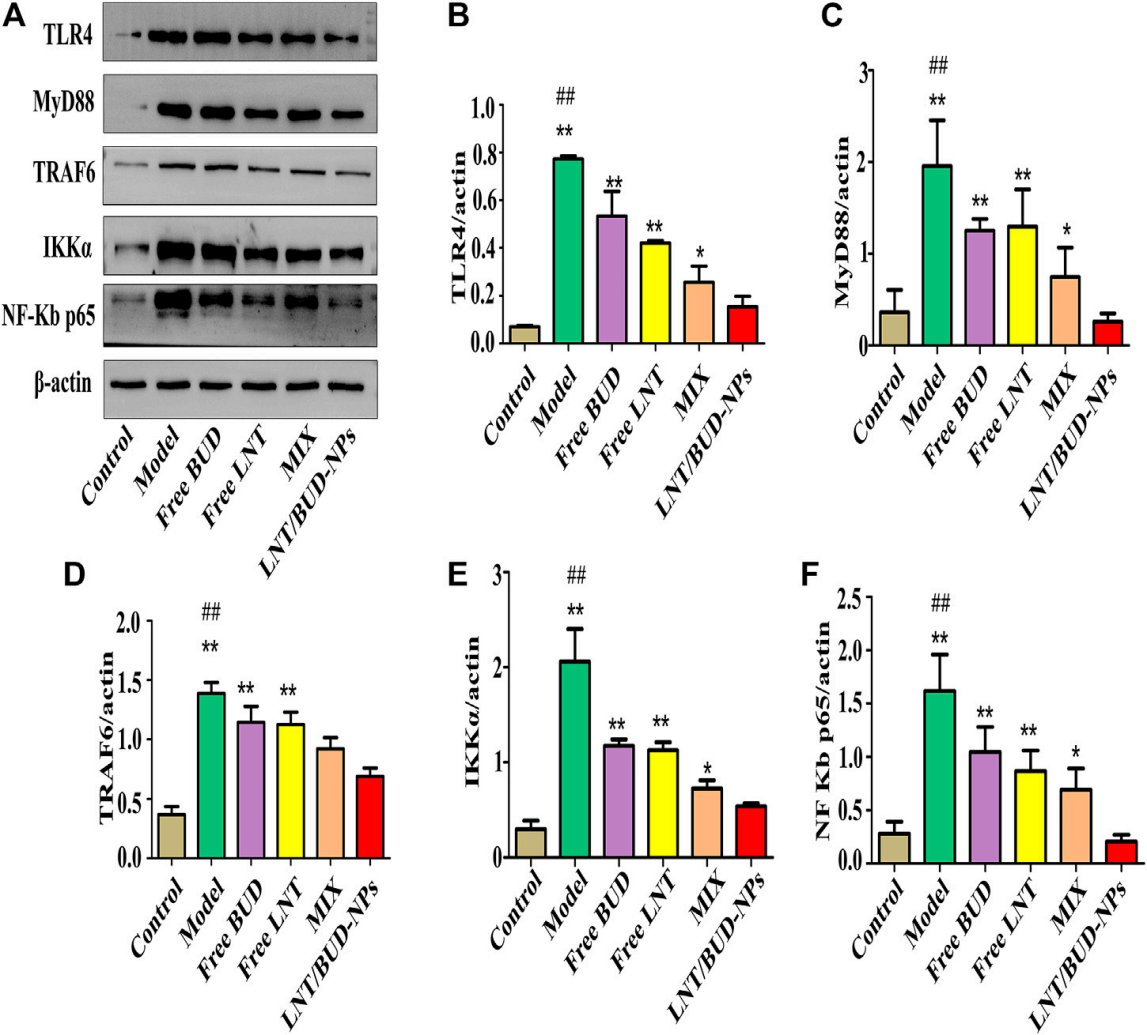
*In vivo* anti-inflammatory efficacy by inhibiting TLR4/NF-κB signaling pathway among the different formulas. **(A)** Western blot images of NF-κB quantify protein concentrations by among the different formulas. **(B–F)** Histogram analysis of TLR4, MyD88, TRAF6, IKKα, and NF-κB p65 expression in the colon among different formulas. ^*^ *p* < 0.05, **p < 0.01 vs. LNT/BUD-NPs; ^#^ *p* < 0.05, ^##^ *p* < 0.01 vs. Model; *n* = 9.

TLR4/NF-κB pathway proteins, such as TLR4, MyD88, TRAF6, IKKα, and NF-κB, were evaluated in this study by Western blot assay as shown in Figure 7A. Quantitative analysis of the different proteins are shown in Figures 7B–F, compared with the Control group, the level of TLR4, MyD88, TRAF6, IKKα, and NF-κB p65 all show clear increases in the Model group (*p* < 0.01), indicating the DSS-induced UC mouse model was valid. As expected, the LNT/BUD-NPs group showed a significant reduction (*p* < 0.05) in all detected proteins compared with the other three drug-treated groups, including Free BUD, Free LNT, and MIX. These data support the validity of using the LNT/BUD-NPs oral delivery system to effectively enhance the anti-UC efficiency *via* TLR4/NF-κB signaling pathway.

## Conclusion

**FIGURE 8:**
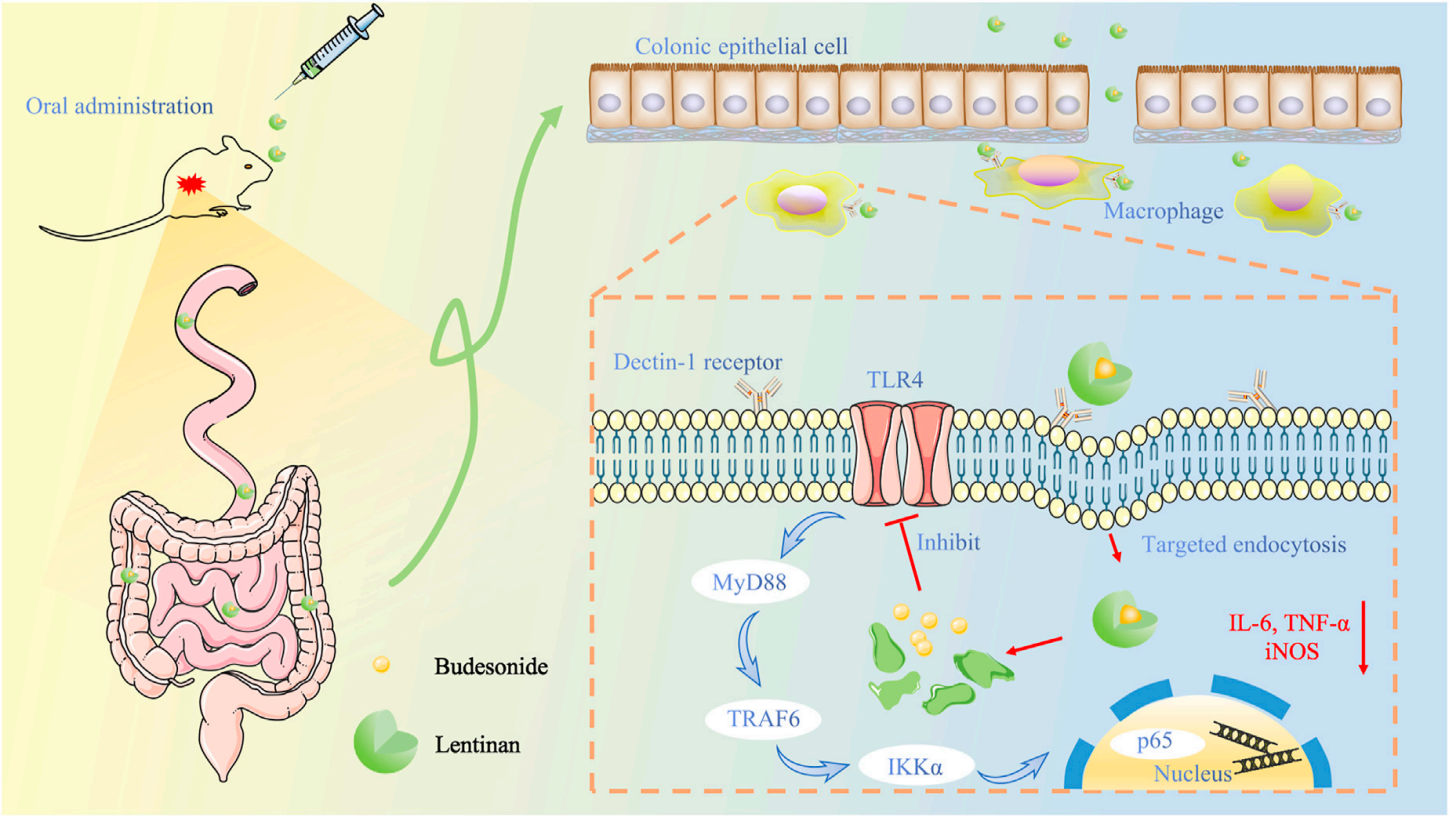
Schematic illustration of lentinan-based oral nanoparticle loaded budesonide with macrophages-targeting ability for treatment of ulcerative colitis.

In this study, we designed an oral delivery system of loaded BUD for UC treatment that was able to target macrophages by β-glucan to Dectin-1 receptors (Figure 8). Our LNT/BUD-NPs have a narrow and stable size distribution, good targeting ability, and UC treatment efficacy. Four contributions of LNT to BUD were confirmed in this study. First, LNT show good loading capacity to BUD which has high EE (92.19%) and LE (9.58%). Second, both uptake analysis and biodistribution evaluation results indicate macrophages-target the ability of LNT directly and indirectly, respectively. Third, LNT/BUD-NPs were stable in SGF confirmed by size and NPs shape changes. Last, the releasing behavior of LNT/BUD-NPs illustrated mouse intestinal content enhances release ability caused by LNT. Collectively, this evidence suggests that LNT is a promising natural oral nano-carrier for treatment of UC.

Compared to other novel nanometers, BUD delivery systems target UC treatment (Naeem et al., 2015; Zhou and Qian, 2018; Krishnamachari et al., 2007; Naeem et al., 2015). As well as the four advantages of LNT mentioned above, two further aspects need to be taken into account. On the one hand, LNT is an abundant polysaccharide with a low price, which is a key factor for industrialization (Zhang et al., 2011). On the other hand, the efficacy of LNT binding to UC, as well as the strong synergistic effects between LNT and BUD are also observed in this study. However, the mechanisms of the synergistic effects need to be researched further. Finally, LNT is an excellent candidate for application in treatment UC and loading of drugs.

## Data Availability

The raw data supporting the conclusions of this article will be made available by the authors, without undue reservation.

## References

[B1] Ali H. Weigmann B. Collnot E.-M. Khan S. A. Windbergs M. Lehr C.-M. (2016). Budesonide Loaded PLGA Nanoparticles for Targeting the Inflamed Intestinal Mucosa-Pharmaceutical Characterization and Fluorescence Imaging. Pharm. Res. 33 (5), 1085–1092. 10.1007/s11095-015-1852-6 26718953

[B2] Amini M. A. Faramarzi M. A. Gilani K. Moazeni E. Esmaeilzadeh-Gharehdaghi E. Amani A. (2014). Production, Characterisation, Andin Vitronebulisation Performance of Budesonide-Loaded PLA Nanoparticles. J. Microencapsulation 31 (5), 422–429. 10.3109/02652048.2013.871358 24697188

[B3] Aratani Y. (2018). Myeloperoxidase: Its Role for Host Defense, Inflammation, and Neutrophil Function. Arch. Biochem. Biophys. 640, 47–52. 10.1016/j.abb.2018.01.004 29336940

[B4] Armuzzi A. Tarallo M. Lucas J. Bluff D. Hoskin B. Bargo D. (2020). The Association between Disease Activity and Patient-Reported Outcomes in Patients with Moderate-To-Severe Ulcerative Colitis in the United States and Europe. BMC Gastroenterol. 20 (1), 18. 10.1186/s12876-020-1164-0 31964359 PMC6975026

[B5] Beloqui A. Coco R. Alhouayek M. Solinís M. Á. Rodríguez-Gascón A. Muccioli G. G. (2013). Budesonide-loaded Nanostructured Lipid Carriers Reduce Inflammation in Murine DSS-Induced Colitis. Int. J. Pharmaceutics 454 (2), 775–783. 10.1016/j.ijpharm.2013.05.017 23694806

[B6] Buhecha M. D. Lansley A. B. Somavarapu S. Pannala A. S. (2019). Development and Characterization of PLA Nanoparticles for Pulmonary Drug Delivery: Co-encapsulation of Theophylline and Budesonide, a Hydrophilic and Lipophilic Drug. J. Drug Deliv. Sci. Tech. 53, 101128. 10.1016/j.jddst.2019.101128

[B7] Dulal H. P. Adachi Y. Ohno N. Yamaguchi Y. (2018). β-Glucan-induced Cooperative Oligomerization of Dectin-1 C-type Lectin-like Domain. Glycobiology 28 (8), 612–623. 10.1093/glycob/cwy039 29897456

[B8] Edsbäcker S. Andersson T. (2004). Pharmacokinetics of Budesonide (Entocort EC) Capsules for Crohn's Disease. Clin. Pharmacokinet. 43 (12), 803–821. 10.2165/00003088-200443120-00003 15355126

[B9] Gani A. Shah A. Ahmad M. Ashwar B. A. Masoodi F. A. (2018). β-d-glucan as an Enteric Delivery Vehicle for Probiotics. Int. J. Biol. Macromolecules 106, 864–869. 10.1016/j.ijbiomac.2017.08.093 28842198

[B10] Gao F. Zhang J. Fu C. Xie X. Peng F. You J. (2017). iRGD-Modified Lipid–Polymer Hybrid Nanoparticles Loaded with Isoliquiritigenin to Enhance Anti-breast Cancer Effect and Tumor-Targeting Ability. Ijn 12, 4147–4162. 10.2147/Ijn.S134148 28615942 PMC5459978

[B11] Gou S. Chen Q. Liu Y. Zeng L. Song H. Xu Z. (2018). Green Fabrication of Ovalbumin Nanoparticles as Natural Polyphenol Carriers for Ulcerative Colitis Therapy. ACS Sust. Chem. Eng. 6 (10), 12658–12667. 10.1021/acssuschemeng.8b01613

[B12] Gou S. Huang Y. Wan Y. Ma Y. Zhou X. Tong X. (2019). Multi-bioresponsive Silk Fibroin-Based Nanoparticles with On-Demand Cytoplasmic Drug Release Capacity for CD44-Targeted Alleviation of Ulcerative Colitis. Biomaterials 212, 39–54. 10.1016/j.biomaterials.2019.05.012 31103945

[B13] Hemsworth G. R. Déjean G. Davies G. J. Brumer H. (2016). Learning from Microbial Strategies for Polysaccharide Degradation. Biochem. Soc. Trans. 44, 94–108. 10.1042/Bst20150180 26862194

[B14] Krishnamachari Y. Madan P. Lin S. (2007). Development of pH- and Time-dependent Oral Microparticles to Optimize Budesonide Delivery to Ileum and colon. Int. J. Pharmaceutics 338 (1-2), 238–247. 10.1016/j.ijpharm.2007.02.015 17368982

[B15] Lee D. H. Kim H. W. (2014). Innate Immunity Induced by Fungal β-Glucans via Dectin-1 Signaling Pathway. Int. J. Med. Mushr 16 (1), 1–16. 10.1615/intjmedmushr.v16.i1.10 24940900

[B16] Liu Y. Liu X. Hua W. Wei Q. Fang X. Zhao Z. (2018). Berberine Inhibits Macrophage M1 Polarization via AKT1/SOCS1/NF-Κb Signaling Pathway to Protect against DSS-Induced Colitis. Int. Immunopharmacology 57, 121–131. 10.1016/j.intimp.2018.01.049 29482156

[B17] Liu Y. Yin H. Zhao M. Lu Q. (2014). TLR2 and TLR4 in Autoimmune Diseases: a Comprehensive Review. Clinic Rev. Allerg Immunol. 47 (2), 136–147. 10.1007/s12016-013-8402-y 24352680

[B18] Luo R. F. Lin M. S. Zhang C. Shi J. F. Zhang S. Y. Chen Q. Y. (2020). Genipin-crosslinked Human Serum Albumin Coating Using a Tannic Acid Layer for Enhanced Oral Administration of Curcumin in the Treatment of Ulcerative Colitis. Food Chem. 330, 127241. 10.1016/j.foodchem.2020.127241 32540526

[B19] Luo R. Lin M. Fu C. Zhang J. Chen Q. Zhang C. (2021). Calcium Pectinate and Hyaluronic Acid Modified Lactoferrin Nanoparticles Loaded Rhein with Dual-Targeting for Ulcerative Colitis Treatment. Carbohydr. Polym. 263, 117998. 10.1016/j.carbpol.2021.117998 33858583

[B20] McKeage K. Goa K. L. (2002). Budesonide (Entocort? EC Capsules)*. Drugs 62 (15), 2263–2282. 10.2165/00003495-200262150-00015 12381231

[B21] Miehlke S. Acosta M. B.-d. Bouma G. Carpio D. Magro F. Moreels T. (2018). Oral Budesonide in Gastrointestinal and Liver Disease: A Practical Guide for the Clinician. J. Gastroenterol. Hepatol. 33, 1574–1581. 10.1111/jgh.14151 29603368

[B22] Mitamura T. Sakamoto S. Suzuki S. Yoshimura S. Maemura M. Kudo H. (2000). Effects of Lentinan on Colorectal Carcinogenesis in Mice with Ulcerative Colitis. Oncol. Rep. 7 (3), 599–601. 10.3892/or.7.3.599 10767375

[B23] Naeem M. Cao J. Choi M. Kim W. S. Moon H. R. Lee B. L. (2015). Enhanced Therapeutic Efficacy of Budesonide in Experimental Colitis with enzyme/pH Dual-Sensitive Polymeric Nanoparticles. Int. J. Nanomedicine 10, 4565–4580. 10.2147/Ijn.S87816 26213469 PMC4509535

[B24] Ng S. C. Kaplan G. G. Tang W. Banerjee R. Adigopula B. Underwood F. E. (2019). Population Density and Risk of Inflammatory Bowel Disease: A Prospective Population-Based Study in 13 Countries or Regions in Asia-Pacific. Am. J. Gastroenterol. 114 (1), 107–115. 10.1038/s41395-018-0233-2 30177785

[B25] Perse M. Cerar A. (2012). Dextran Sodium Sulphate Colitis Mouse Model: Traps and Tricks. J. Biomed. Biotechnol. 2012, 718617. 10.1155/2012/718617 22665990 PMC3361365

[B26] Rubin D. T. Ananthakrishnan A. N. Siegel C. A. Sauer B. G. Long M. D. (2019). ACG Clinical Guideline: Ulcerative Colitis in Adults. Am. J. Gastroenterol. 114 (3), 384–413. 10.14309/ajg.0000000000000152 30840605

[B27] Vetvicka V. Vannucci L. Sima P. (2020). Beta-glucan as a New Tool in Vaccine Development. Scand. J. Immunol. 91 (2), e12833. 10.1111/sji.12833 31544248

[B28] Wang F. Wang K. Xu W. Zhao S. Ye D. Wang Y. (2017). SIRT5 Desuccinylates and Activates Pyruvate Kinase M2 to Block Macrophage IL-1β Production and to Prevent DSS-Induced Colitis in Mice. Cel Rep. 19 (11), 2331–2344. 10.1016/j.celrep.2017.05.065 28614718

[B29] Yen H.-H. Weng M.-T. Tung C.-C. Wang Y.-T. Chang Y. T. Chang C.-H. (2019). Epidemiological Trend in Inflammatory Bowel Disease in Taiwan from 2001 to 2015: a Nationwide Populationbased Study. Intest Res. 17 (1), 54–62. 10.5217/ir.2018.00096 30449079 PMC6361021

[B30] Zhang Y. Li S. Wang X. Zhang L. Cheung P. C. K. (2011). Advances in Lentinan: Isolation, Structure, Chain Conformation and Bioactivities. Food Hydrocolloids 25 (2), 196–206. 10.1016/j.foodhyd.2010.02.001

[B31] Zhao X. Zhou C. Ma J. Zhu Y. Sun M. Wang P. (2017). Efficacy and Safety of Rectal 5-aminosalicylic Acid versus Corticosteroids in Active Distal Ulcerative Colitis: a Systematic Review and Network Meta-Analysis. Sci. Rep. 7, 46693. 10.1038/srep46693 28440311 PMC5404224

[B32] Zhou H. Qian H. (2018). Preparation and Characterization of pH-Sensitive Nanoparticles of Budesonide for the Treatment of Ulcerative Colitis. Dddt 12, 2601–2609. 10.2147/Dddt.S170676 30174414 PMC6110634

[B33] Zhou Q. Feng Y. Liu N. Hu J. Yang F. WU L. (2020). Determination of the Effect of Atomization Temperature on the Stability of Inhaled Budesonide Suspension by RP-HPLC Method. Chin. Med. Equipment 17(1), 160–162. 10.3969/J.ISSN.1672-8270.2020.01.042

