# Supplementary figures and images for: Lentinan-Based Oral Nanoparticle Loaded Budesonide With Macrophage-Targeting Ability for Treatment of Ulcerative Colitis
Source: Front Bioeng Biotechnol. 2021 Aug 27;9:702173. doi: 10.3389/fbioe.2021.702173 (PMC8429481; doi:10.3389/fbioe.2021.702173)

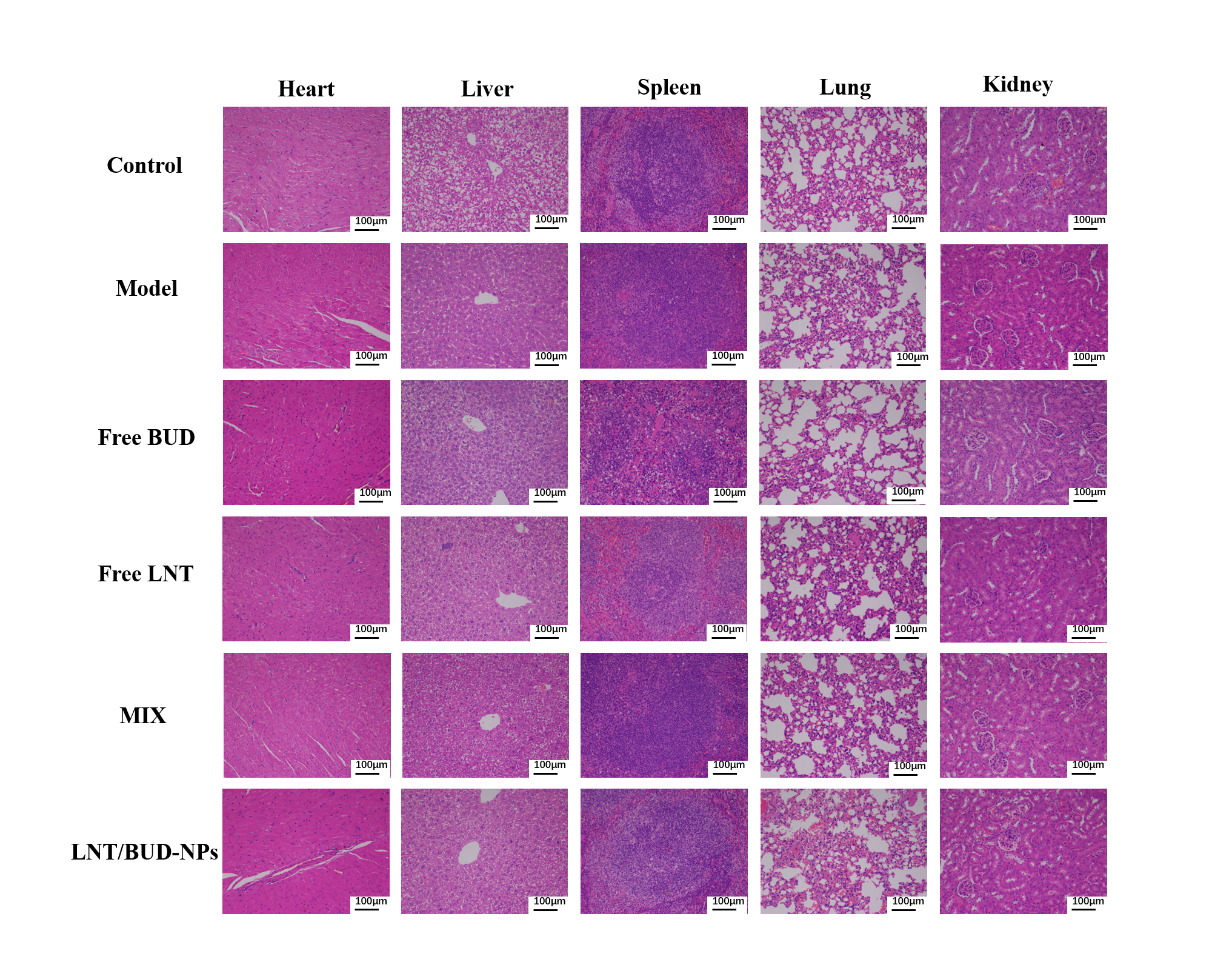

Supplement: Supplementary file 1 [file Image1.TIF]
